# A Think Aloud Study Comparing the Validity and Acceptability of Discrete Choice and Best Worst Scaling Methods

**DOI:** 10.1371/journal.pone.0090635

**Published:** 2014-04-23

**Authors:** Jennifer A. Whitty, Ruth Walker, Xanthe Golenko, Julie Ratcliffe

**Affiliations:** 1 Griffith Health Institute and the Centre for Applied Health Economics, School of Medicine, Griffith University, Logan, Australia; 2 Southgate Institute for Health, Society and Equity, Flinders University, Adelaide, Australia; 3 Flinders Clinical Effectiveness, School of Medicine, Flinders University, Adelaide, Australia; Newcastle University, United Kingdom

## Abstract

**Objectives:**

This study provides insights into the validity and acceptability of Discrete Choice Experiment (DCE) and profile-case Best Worst Scaling (BWS) methods for eliciting preferences for health care in a priority-setting context.

**Methods:**

An adult sample (N = 24) undertook a traditional DCE and a BWS choice task as part of a wider survey on Health Technology Assessment decision criteria. A ‘think aloud’ protocol was applied, whereby participants verbalized their thinking while making choices. Internal validity and acceptability were assessed through a thematic analysis of the decision-making process emerging from the qualitative data and a repeated choice task.

**Results:**

A thematic analysis of the decision-making process demonstrated clear evidence of ‘trading’ between multiple attribute/levels for the DCE, and to a lesser extent for the BWS task. Limited evidence consistent with a sequential decision-making model was observed for the BWS task. For the BWS task, some participants found choosing the worst attribute/level conceptually challenging. A desire to provide a complete ranking from best to worst was observed. The majority (18,75%) of participants indicated a preference for DCE, as they felt this enabled comparison of alternative full profiles. Those preferring BWS were averse to choosing an undesirable characteristic that was part of a ‘package’, or perceived BWS to be less ethically conflicting or burdensome. In a repeated choice task, more participants were consistent for the DCE (22,92%) than BWS (10,42%) (p = 0.002).

**Conclusions:**

This study supports the validity and acceptability of the traditional DCE format. Findings relating to the application of BWS profile methods are less definitive. Research avenues to further clarify the comparative merits of these preference elicitation methods are identified.

## Introduction

Choice-based methods represent a fundamental approach for eliciting stated preferences for the health and non-health related characteristics of health care [1]. Features contributing to the popularity of these methods from a health economics perspective include their firm basis in economic theory and the requirement to explicitly consider trade-offs in the determination of preferences [2]. Stated preferences methods are used to derive important inputs for use in economic evaluation in health care, including utility indices to weight quality of life according to preferences for living in different health states [3], [4]. Researchers are also investigating the potential role for stated preference methods in developing multi-criteria or distributional weights for use in priority setting [5], [6].

The traditional discrete choice experiment (DCE), which asks respondents to choose between two or more whole profiles described by a number of attribute levels, is one of the most established choice-based formats used to elicit stated preferences in health [1]. However, more recently, best worst scaling (BWS) has developed as an alternative format for preference elicitation [7]. There are three subtypes of BWS; in all types, respondents are asked to choose the best/worst (or most/least) from a number of options [8]. In object case BWS (also referred to as type 1), the choice is between different whole objects which are not described by distinct attributes. For example, a respondent might indicate the most and least important principle for health reform [9]. In profile case BWS (type 2) the choice is between individual attribute/level pairs within a single profile. Thus, the respondent is shown a single profile (e.g. of a health state) consisting of a number of attribute/level combinations (e.g. levels of each health domain in the health state) and is asked to choose the best and worst attribute/level (e.g. health domain level) in the profile [3], [10]. In the multiprofile case (type 3), the choice is between whole profiles, where each profile is described by a number of attribute/levels (e.g. between a number of different health states profiles, each described by a number of domain levels). Thus, multiprofile BWS is akin to the traditional DCE with a minimum of three profiles in each choice set, and in this situation elicits a more complete preference ranking (best/most preferred and worst/least preferred) across profiles in each set compared to the traditional DCE format (which would only elicit the most preferred).

The DCE and BWS methods share a number of attractive features; both require only an ordinal assumption of preference structure and are grounded in the same random utility framework [8], [11], [12]. However, proponents for the BWS method argue it potentially obtains more information from respondents whilst exposing them to a lower level of burden [8]. This may in turn have a beneficial impact on data quality and response rate. Further, the profile type BWS has a potential advantage particularly for studies involving qualitative attributes as it can elicit preference weights to indicate attribute impact distinct from the impact of attribute levels [7]. Despite these perceived advantages, applications of the BWS approach in the health care sector have been relatively limited to date, and understanding of this method remains in an early growth phase. Very few studies have compared DCE and BWS approaches [4], [13], [14] and issues such as respondent burden and acceptability have not been directly compared previously.

Researchers have promoted the importance of qualitative work to not only develop preference based instruments but also explore methodological validity and the underlying rationale for responses in the health preference literature [15]–[18]. Two previous studies have demonstrated the utility of the ‘Think Aloud’ approach whereby participants verbalize their thinking whilst making choices in the traditional DCE context [19], [20].

The aim of this study was to apply the think aloud approach to compare the validity and acceptability of the traditional DCE and the profile case BWS methods for eliciting preferences for health care services and interventions. The profile case BWS is chosen as it is the most applied BWS format in health care [8], with previous applications in health service research [21] as well as health state valuation [4], [7], [22]. It has been suggested that the profile case should be used where computational burden for respondents in answering a traditional DCE is potentially too high [8].Specifically, within a priority setting context this study sought to provide insights into:

1. Internal validity through (i) an assessment of the decision approach that participants take when making choices for each method and (ii) an internal consistency test; and
2. The acceptability of the methods from the participants' perspective.

## Methods

### 1. Ethics Statement

Ethical approval for the study was provided by the Griffith University Human Research Ethics Committee. An invitation to participate was emailed to all staff and students at a university campus in Queensland, Australia. Participants provided written informed consent to participate in the study, and the ethics committee approved this consent procedure. The informed consent reassured confidentiality and that only the research team would have access to the audiotape transcription. Therefore, to maintain ethical integrity, we are unable to deposit the data in a repository for public access. Nevertheless, if readers wish to access the data it may be possible to obtain ethical approval for access to de-identified data on an individual case by case basis (interested readers should contact the authors).

### 2. Survey

The survey was designed as part of a broader study to assess public preferences surrounding criteria that might be used to make decisions around the funding of new health technologies. Attributes and levels for the choices were derived based on a review of the literature on public preferences in a priority setting contexts [23]–[25], decision-making criteria that might be used in health technology assessment (HTA) [26]–[30], and refined following semi-structured interviews with a public convenience sample (n = 19) (Table 1).

**Table 1:** Attributes and levels for the DCE and BWS tasks.

Attribute	Definition	Levels
BENEFIT	What is the main benefit from the intervention?	Prevents people becoming ill
		Diagnoses illness early
		Treats people when they become ill, resulting in an improvement in quality of life
		Treats people when they become ill, resulting in a one year increase in survival
		Reduces the risk of suffering a side effect from treatment
		Reduces hospital waiting times
VALUE	Is the intervention expected to provide good value for money?	Yes
		No
NEED	Is there already an alternative intervention available for this purpose?	No alternative intervention is available for this purpose
		An alternative but different intervention is already available for this purpose
		This is an upgrade of an existing intervention
BURDEN	How many patients in Queensland are expected to benefit from this intervention each year?	10
		500
		1000
		2000
AGE	On average, how old are the patients?	10 years
		35 years
		60 years
		85 years
EQUITY 1	Does the intervention address a particular need for indigenous Queenslanders?	Yes
		No
EQUITY 2	Does the intervention address a particular need for Queenslanders living in rural or remote areas?	Yes
		No

**Figure 1:**
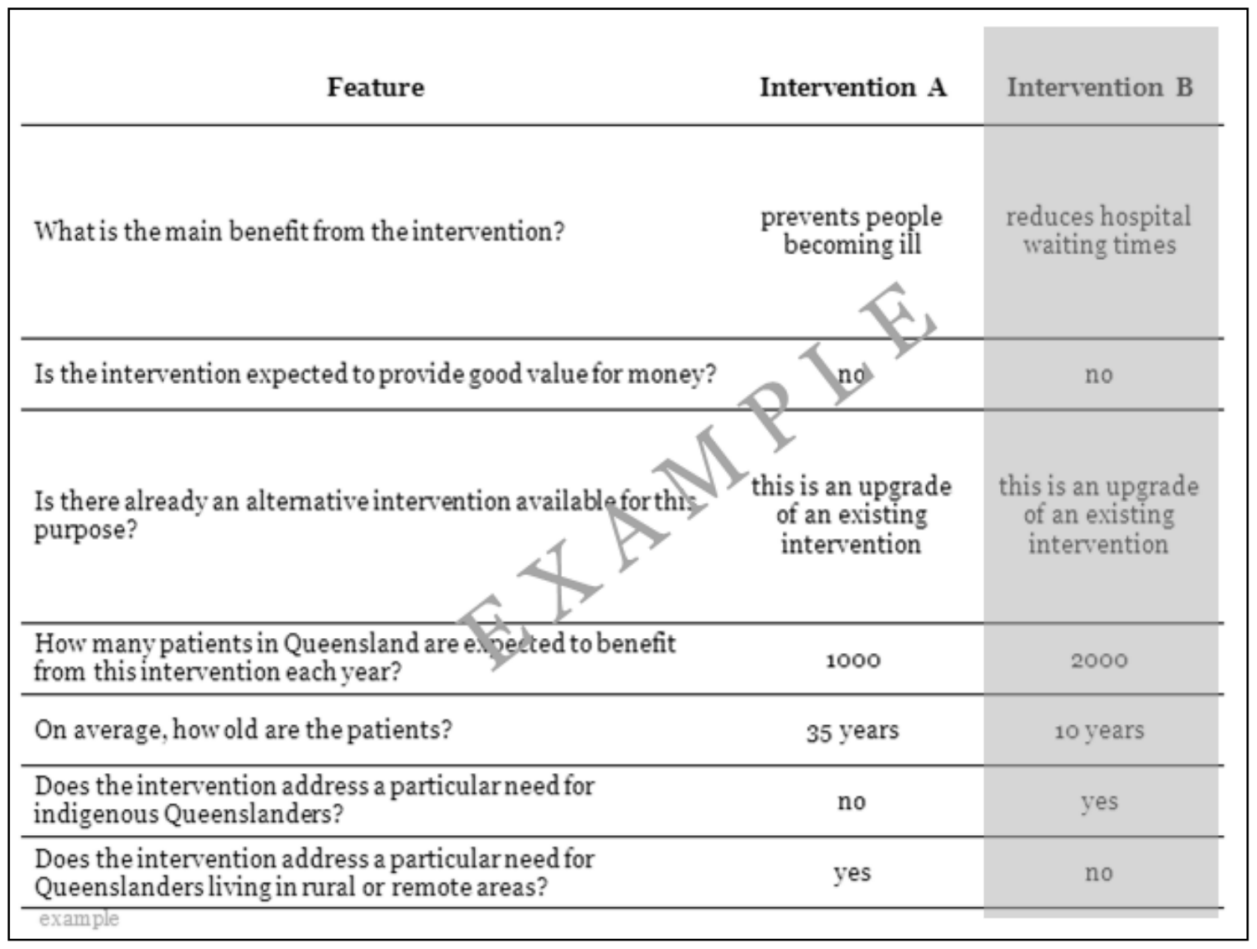
Illustrative example of DCE task.

**Figure 2:**
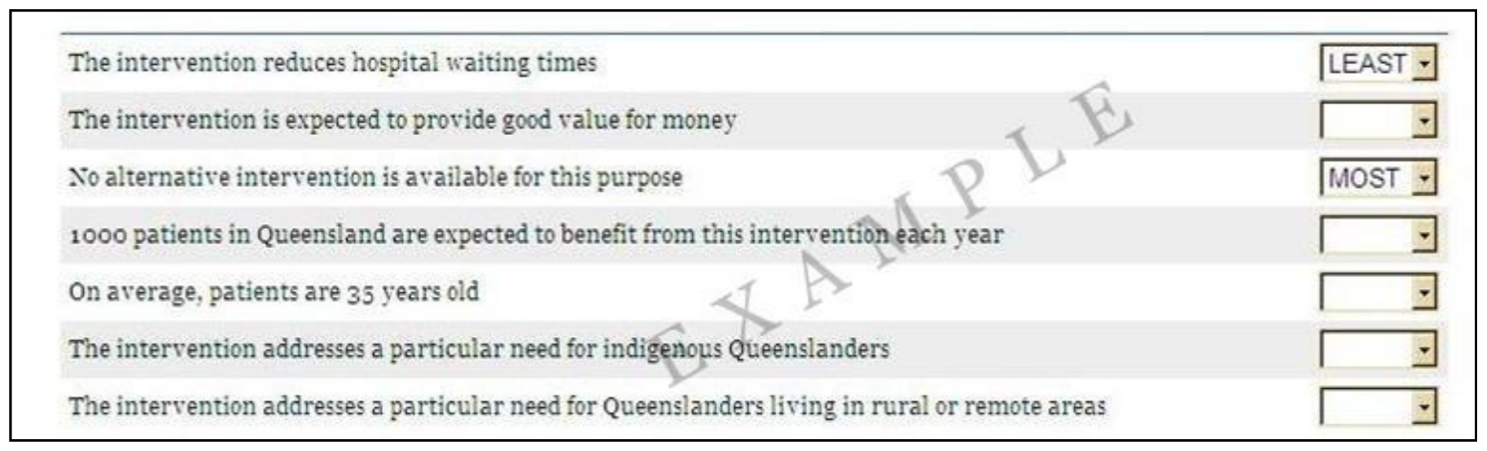
Illustrative example of BWS task.

An orthogonal design consisting of 15 columns (seven columns for the attributes, each with two alternatives, and one additional blocking column) and 72 rows was selected using NGENE software [31]. The DCE task was presented as two alternative profiles specified by the first 14 columns of each row. Participants were asked to choose which of the two complete profiles they would prefer to be funded. The BWS task was presented as a series of single profiles specified using the first seven columns. Participants were asked to choose which of the seven attribute and level combinations in the profile they considered to be the most and least important consideration for a funding decision. An illustrative DCE and BWS task is presented in Figures 1 and 2.

To ensure the survey was manageable the 72 rows were divided into twelve blocks of six rows. This was achieved using the fifteenth column in the design, which had twelve levels (one associated with each “block”). Each of twelve survey versions contained one DCE block randomly paired with one different BWS block. To control for possible ordering bias, six versions presented the DCE block first while the remaining six presented the BWS block first. A number of sociodemographic questions were also included, which were always presented last.

### 3. Sample

Interviews were undertaken with 24 participants, who were sequentially assigned to complete one of the twelve survey versions. Participants were recruited from a university campus. All staff and students (aged 17 years and over) affiliated with the campus were sent an email invitation to participate. In addition, flyers advertising the study were posted on campus noticeboards. Those expressing interest were invited sequentially, until 24 interviews were completed. A sample size of 24 was chosen as this ensured every version was completed by two participants. Preliminary thematic analysis of the data as the interviews progressed also indicated that data saturation had been reached. Participants were offered AU$25 cash to compensate them for their time.

### 4. ‘Think Aloud’ Interview

Interviews were conducted in a private office at the university campus in November 2011. The researcher explained the study, informed consent was obtained and the interview was audio-recorded. A warm-up exercise (noughts and crosses) was undertaken to familiarise participants with the process of thinking aloud [20]. Participants then completed the survey online in the presence of the researcher, who sat behind them to avoid interruption or distraction. Participants were instructed to think aloud concurrently whilst completing the choice-based questions in the survey. Verbal protocol analysis was used to guide the think aloud approach [32]. If after the first question, or any two subsequent questions, the participant had not verbalised their thought processes, the interviewer was instructed to prompt the participant “If you could keep thinking aloud …..”. At the conclusion of the interview, participants were asked which of the two choice tasks they preferred and why.

### 5. Data analysis

Consistent with the study aim, the think aloud data were assessed thematically [33] to identify the decision making *process* i.e how participants made their decisions using the two different tasks (DCE, BWS) as opposed to understanding the *content/context* of the rationale on which they were basing their decisions. An initial coding framework was developed by a researcher with expertise in choice-based preference methods (JW). To ensure consistency all transcripts were coded in NVIVO software [34] by one researcher (XG), expanding the coding framework for further themes that emerged from the data. To ensure coding was comprehensive and reliable, a third researcher (RW) independently cross-checked the coding for a random sample of the data (six interviews) against the framework. Data relating to a consistency check and the final question relating to preference for method at the end of the interview were analysed descriptively.

### 6. Assessing internal validity

Both the traditional DCE and BWS methods are derived from random utility theory which is based on several assumptions related to rational consumer choice behaviour [20]. To assess internal validity, the qualitative think aloud' data were explored seeking evidence to support these assumptions.

Firstly, the traditional DCE method assumes continuity of preferences; that is, individuals can be compensated for a reduction in one desirable characteristic by an increase in another, even at extreme limits [20], [35]. This is reflected in decision-making as trading between attribute levels, across profiles. The profile case BWS assumes trading to a lesser extent [4], [8], since participants are only required to choose a best and worst attribute level within a single profile. Further, for the BWS task there is not an explicit opportunity cost. Thus, evidence of compensatory decision-making or trading between multiple attributes/levels was sought to support the assumption of preference continuity, but was anticipated to be likely to be exhibited to a greater extent for the traditional DCE than profile case BWS task. Within the think aloud data, trading was considered to be occurring whenever participants indicated they would consider forgoing one attribute and/or level in favour of choosing another (either between profiles for the DCE or within a profile for the BWS task).

In addition, for the BWS task, the approach participants take to choosing the best and worst option is an important consideration for validity of potential analysis methods. A number of possible psychological decision-making models have been outlined including sequential models (e.g. best then worst chosen, or vice versa) [36]–[38] and the paired maxdiff model [36], [38], [39] where all possible best/worst pairs in the choice are compared and the pair with the maximum difference on the underlying latent utility scale is chosen [8], [21]. The approach to analysis should theoretically be consistent with the underlying psychological choice model [8]. Yet, thus far only supposition suggests which decision approach is applied by respondents to BWS tasks. Insights into the use of a sequencing or maxdiff style approach were sought from the data.

Finally for both tasks, the assumption of completeness was assessed using an internal consistency test. Completeness implies that participants have a well defined preference between competing alternatives [20], [35]. In each block one profile was repeated for the DCE and BWS tasks. Thus, each participant was asked to complete a total of seven DCE and seven BWS tasks. Participants were deemed to have passed the consistency test if they gave the same choice response for the repeated DCE tasks and chose the same attribute/level combination as most and least important for the repeated BWS tasks. Passing the consistency test would indicate the participant's preferences were complete and they answered the survey in a logical manner.

## Results

Think aloud participants had a mean age of 36.6 years (SD 10.5 range 18 to 56 years), and 19 (79%) were female. On average, the think aloud interview lasted 38 minutes (SD 10, range 25 to 60 minutes). After clear instructions on the requirement to think aloud were provided by the interviewer, only one participant (ID 12) required prompting to keep thinking aloud during the interview. One further participant (ID 18) expressed that they found the think aloud task “awkward” at the end of the interview.

### 1. Thematic Analysis of Decision Process from Think Aloud data

Five main themes describing the decision process that appeared to be employed by participants were identified (Table 2). These are presented separated by task, and the similarities and differences between the DCE and BWS approaches suggested from the data are compared and contrasted.

**Table 2:** Themes describing decision process and method for which they were observed.

Theme:	Observed for:
Trading	DCE and BWS
• Evidence participants were comparing between alternative profiles and trading between the attribute/levels across alternatives was strongly exhibited for the DCE task.	
• Evidence for trading between attribute/levels within a profile was weakly exhibited for the BWS task.	
Psychological decision model	BWS
• Limited but inconsistent evidence was observed supporting a sequential decision-making model.	
• No evidence was observed supporting a maxdiff decision model.	
Difficulty conceptualizing ‘least’ important	BWS
• Some participants found it challenging to choose a least important attribute/level, when even the least important might still be perceived as either still important, or conversely as not important at all, for a funding decision.	
Desire to rate	BWS
• Some participants wanted to rate or rank attribute/levels to provide a more complete preference ordering.	
Lack of variation	BWS
• Some participants showed a lack of variation in their choices, consistently choosing the same attribute/level as least important for all profiles.	

### 1.1 Discrete choice task

#### Trading

The DCE task elicited many comments indicating participants were comparing the two alternatives in the choice set. Consistent with the theoretical assumptions underlying the DCE method, many participants indicated they were trading between the levels of multiple attributes across the two alternatives. This was exemplified by one participant who stated:

*“A versus B, diagnose early versus increase in survival, … they are both cost effective, one has an alternative, one has no alternative, … both the same population size, same age, one is indigenous whereas the other one is rural and remote. Intervention B for early diagnosis, go with intervention B.”* [ID 15]

One further participant gave a detailed insight into their thought processes, but evidenced also the challenges associated with trading attributes resulting in an opportunity cost:

*“Reducing a side effect here, there is an existing intervention here, there is no alternative here, it is more expensive, it is older people, it doesn't help rural, 25 year age gap ….. Upgrade, no alternative, twice as many people. … this is going to help rural people, twice as many people but there is already an intervention. This is good value for money, reducing the risk of suffering a side effect. … I think I am going to go here because they don't have an alternative. I am not happy because of this and this.”* [ID 24]

Interestingly, thoughts expressed by one participant suggested that rather than trading the difference in attribute levels across profiles, they counted how many attributes had a preferred level in each profile in a dichotomised fashion and then compared this number. This would infer a violation of the conventional linear additive model used to specify DCE choice models.

*“Right, so the first two are pretty much the same, prevents from becoming ill, diagnose early, not good value for money, … A gets a tick for value for money, it gets a tick for having no alternative and a tick for having 1000 patients, I suppose if they are 60 versus 35, it is probably better if you are preventing illness, … that is one tick for intervention B. Two ticks for intervention B and 3 ticks for intervention A, so I am going to have to go with A.”* [ID 22]

One participant expressed ethical challenges with making a choice between the two alternatives and indicated that a points or weighting system for different attributes might assist them to make that choice:

*“It is the ethical stuff … Do you choose the younger people, should it be based on that, should it be based on the fact there is no alternative available, should it be based on the fact that it helps rural or indigenous people, you almost need a criteria that gives them points maybe.”* [ID 24]

### 1.2 Best worst task

#### Trading

Some participants indicated they were considering a trade-off between more than one attribute/level within each BWS profile, to decide which characteristics were the most or least important within that profile. For example:

*“I think the most important is the intervention prevents people from becoming ill and the least important is I am tossing up between on average patients are 60 years old or doesn't address a particular need for Queenslanders living in rural or remote areas.”* [ID 23]

Consistent with the BWS task, and in contrast to the DCE, participants didn't have to consider forgoing a complete intervention package in favour of another. Thus, the opportunity cost of their decision was an attribute/level not a profile, and arguably could be viewed to be less meaningful.

The same participant who expressed a desire for a weighting system for the DCE task also expressed a desire to have a weighting system to assist with choice of criteria in the BWS task:

*“…the most important reason to fund the intervention. OK, I would almost need to give these a point system or something, in order to make a rational decision with each one, otherwise I am just jumping around.”* [ID24]

#### Psychological decision model

It was difficult to discern any uniform choice approach for the BWS task from the think aloud data. Consistent with a sequential approach, half of participants (n = 12) generally paused, then selected the “most” followed by “least” preferred attribute/level. A smaller number of participants (n = 4) considered all attribute/levels before proclaiming which they felt was most and least important in sequential order or seemed inconsistent in their approach, selecting either most or least preferred in a random order for different profiles (n = 3). The issue of question framing might be important here, in that the question participants were asked in the survey inferred an order (i.e. “Which one of these seven features would rate as the most important reason and which one would rate as the least important reason to fund the intervention?”) which some participants clearly adhered to. However other participants elected to choose either the most or least first which was permitted by the programming for the survey. No evidence was observed to suggest any participant simultaneously compared the most and least attribute/levels in pairs, as would be required for a paired maxdiff approach when making their choices.

#### Difficulty conceptualizing ‘least’ important

Conceptually, some participants found it difficult to determine the least important reason for funding. They appeared to suggest that choosing “least” important was difficult when even that attribute might be a beneficial reason for funding. For example, one participant questioned:

*“… most important reason to fund the intervention, that is easy to understand, but the least important reason is the one they wouldn't really look at? … So they shouldn't worry about that category when they make a decision?”* [ID 16]

Another participant stated:

*“… the least important can be that one …it throws me out a bit…Because it is important.”* [ID1]

Conversely, some also indicated difficulty expressing an attribute/level combination as “least important” when they considered that attribute/level was *not important at all*. One participant stated:

*“I guess we are saying least important but you are still saying it is important and that is a factor but it is the least important factor whereas this issue is completely irrelevant …. but if you put that as least important it is almost like you are in some way saying that this is an issue.”* [ID 22]

This challenge appeared to be related to the least important attribute/level; similar difficulties choosing the most important attribute/level were not observed.

#### Desire to rate

Some participants indicated a desire to rate or rank attribute/level combinations and provide a more complete preference ordering from best to worst, rather than just to choose just one best and worst option. These participants felt restricted by only being able to select one, particularly as most important, in each profile. For example, one participant stated:

*“…I only have 2 choices here - except I would expect to rate them 1–7 … like on a declining importance … I don't think I have got enough choice only being able to respond to 2, most and least.”* [ID 2]

#### Lack of variation

Comments from two participants indicated a lack of variation between profiles in their choice of least important:

*“Again, I look at that is least important, again … indigenous.”* [ID 1]

*“Definitely preventing illness is the most important, I am going to have this money thing as the least in each one.”* [ID 2]

In the extreme, participants consistently choosing the same best or worst attribute/level would result in an inability to identify preference weights for the attribute/level combinations that were not chosen.

### 2. Descriptive comparison of task preference and consistency

A substantially larger proportion of participants stated they preferred the DCE task (18, 75%) than the BWS task (6, 25%). Further, on a Likert scale where 1 represents very easy and 5 very difficult, participants expressed a slightly lower level of difficulty in completing the DCE (mean 3.04 SD 0.95 median 3 range 2 to 5) than the BWS task (mean 3.54 SD 1.02 median 4 range 2 to 5; paired T-test for difference in means p = 0.01). Comments from participants when asked which task they preferred were often intertwined with considerations of the ease or difficulty with which they were able to complete the tasks.

### 2.1 Preference for DCE task

Overwhelmingly, participants expressing a preference for the DCE task indicated they liked having a comparison side by side, and that this assisted their decision task. This was summarised by one participant:

*“It* [the DCE] *gives you all of the information that you need, side by side … for me a lot easier and a lot quicker.”* [ID 23 prefer DCE]

Another stated:

*“I liked the comparison of A to B, so 1000 to 2000 sort of thing, … to have them both set out similar, all next to each other as well helps.”* [ID 10 prefer DCE]

Two participants also emphasised the importance of having the whole profile available for decision-making to support their judgement:

*“…* [I prefer DCE] *because you could look at it in its entirety, could consider the entire as a whole … they can be complex decisions, it seems an easier way for me.”* [ID 5 prefer DCE]

*“I much preferred* [DCE] *because it gave you a chance to explore other reasons; where as in* [BWS] *pretty much money was always my least one… when you have the whole picture, it was easier to make a judgement.”* [ID 6 prefer DCE]

Consistent with this, a number of participants expressed difficulty in making a decision in the BWS task, because they did not have a comparison to make a judgement against.

*“… I think this one was harder … to choose my best and worst … because I didn't have the comparisons.”* [ID 11 prefer DCE]

One participant found *“having to graduate most and least”* in the BWS task too *“subjective”* [ID 13 prefer DCE]. Similarly, another participant, although they preferred the BWS task, indicated that they found the DCE task more *“real”* and less “*abstract*” [ID 24 prefer BWS].

Consistent with the findings from the think aloud analysis, some participants preferring the DCE task expressed difficulty with having to choose just one attribute/level combination that was most/least important in the BWS task.

*“It wasn't just one that was most important in any of them …”* [ID 1 prefer DCE]

*“I think it was just clearer, you could weigh up multiple things at the same time* [with DCE] *rather than forced to pick 2 things* [with BWS].*”* [ID 22 prefer DCE]

### 2.2 Preference for BWS task

Participants preferring the BWS task mentioned a number of reasons for doing so. Three participants indicated they disliked the DCE because they felt forced to choose an attribute/level they didn't agree with because it was part of the whole package. This was eloquently expressed by one participant who stated:

*“For the first lot of questions* [DCE], *… there were some things that I didn't quite agree with in some columns maybe or wasn't concerned with but when I ticked that column I had to choose those anyway because they were in that column…Whereas with this one* [BWS], *value for money, I could just ignore it, it is a consideration but it is not most nor least, so I didn't have to make a judgement.”* [ID 16 prefer BWS]

Interestingly, this participant suggests they disliked the DCE task as it forced them to make a judgement on attribute/level combinations that they didn't feel strongly about. Whilst this might be challenging for participants, it is important for an efficient preference model, since the most challenging questions where the level of utility is balanced between profiles provide the most statistically efficient information [40]. Conversely in a BWS model, if a participant never chooses the attribute/level combinations to which they are indifferent as best or worst, these attribute levels cannot be valued on the underlying latent utility scale.

Two participants preferring the BWS task also suggested it was less burdensome than the DCE task:

*“Both of them are good but you know if you are going to go through a lot going most to least would be the way.”* [ID 4 prefer BWS]

*“*[BWS] *was easier because I was just more familiar with what the questioning framework was like not so much the way in which it was questioned.”* [ID 3 prefer BWS]

One participant indicated they preferred BWS as they “*felt less conflicted ethically*” than with the DCE task [ID 24 prefer BWS], possibly because they were being asked to choose between two groups of people (i.e. there was an explicit and meaningful opportunity cost). This was the same participant whose think aloud data indicated they would like a weighting system to assist them to deal with the ethical challenges implicit in their choices.

### 2.3 Tests for consistency

A substantially greater proportion of participants passed the consistency test for the DCE task (22,92% of participants) than for the BWS task (10,42%) (McNemar's test p = 0.002). For the BWS task, there was no discernable pattern as to whether the inconsistent responses primarily resulted from the best or worst level in the task (5 participants inconsistent for “most”, 5 for “least”, and 4 for both).

## Discussion

This study represents the first qualitative analysis, using a think aloud approach, to compare the DCE and BWS methods. We found the think aloud method to be a useful approach in this context, with prompting seldom required by the interviewer. The exploration revealed some important insights into the decision approaches utilised by participants to each of the two choice-based methods presented. With the DCE there was clear and substantial evidence of comparison between profiles and trading between multiple attributes and levels in the choice process. This is consistent with the underlying assumptions of utility theory, specifically continuity, on which the DCE is based. Interestingly, the observation of ‘dichotomised trading’ in one participant would violate the conventional linear additive specification commonly employed in a DCE analysis. However, the existence of this decision-making approach could be tested in analyses of discrete choice data to see if it explains a greater proportion of the variation in the decision than the more conventional modelling approach. In general however, the DCE participants indicated a decision process that was consistent with underlying theory.

Consistency with underlying theory for the profile case BWS task was a little less clear in this priority-setting context. There has been some debate in the literature on the appropriate decision model to use for BWS analysis [8], [37]. In this BWS design a sequential model would assume participants consistently choose their best from the seven attribute/level alternatives within a profile, and then their worst from the remaining six (or vice versa) [8]. Conversely, a maxdiff model would assume that participants consider all possible pairs of best and worst attribute levels in each profile, and then choose the pair that maximizes the difference in utility (measured on a latent scale) between the best and worst attribute levels [8]. We did not observe sufficient data that would suggest a consistent decision-making approach for the BWS task and therefore we cannot draw definitive conclusions about the internal validity of the BWS task from this exploratory study. Nevertheless, whilst we observed some evidence of sequential decision-making for the BWS task, we did not observe any evidence of a paired maxdiff style decision-making approach. This suggests that the paired maxdiff is not an optimal psychological model on which to base the analysis of BWS data [8].

The data highlight several important considerations related to the application of profile case BWS. Firstly, and interestingly, some participants expressed a desire to rank or rate the attribute/level combinations rather than select only one most and one least preferred. This may suggest that a continuous approach (next most preferred, next least preferred etc) to gain a complete ranking for each profile might be more acceptable for participants. This is in accordance with Flynn's [8] argument that it is preferable to elicit a complete preference ordering from respondents in a choice experiment, and a previous application of this approach to estimate individual preference models using multiprofile case BWS [37]. On the basis of this think aloud study, we would also encourage a full ranking approach to be considered for BWS studies planning an aggregate level analysis.

This study also highlights several areas that require further research to clarify their potential impact on the utility of the profile-based BWS approach. Whilst some participants appeared to consider multiple attribute/level combinations, there was evidence that at least some participants were consistently selecting the same attribute/level combination particularly as worst across multiple BWS questions. This observation might indicate a lack of variation in choice for the BWS approach in estimating preferences for all attribute/levels in this priority-setting context, which would potentially have a negative impact on the efficiency of the decision-making model, or the capacity for parameter weights to be identified for all attribute/level combinations. A larger quantitative study is planned to further test this observation empirically. Secondly, several participants expressed some uncertainty around the requirements of the task to select ‘least’ preferred; this might be a framing issue specific to this survey or the priority setting context.

Perhaps of greater methodological implication is the higher response inconsistency observed for the BWS than DCE task. The potential implications of this for model inference requires exploration; however, it is plausible this may indicate greater randomness in decision-making with the BWS task when applied within this specific priority setting context and might result in a preference model with a lower explanatory and prediction power for the BWS than the DCE data. Such a finding would be consistent with the greater acceptability of the DCE than BWS for the majority of participants in this study, but inconsistent with claims for the potential benefits of BWS over the DCE, which include that the BWS task is likely to lead to less respondent burden and randomness in decision-making [4], [8]. Reasons for this trend were not explored in this study. It may be that the questions raised by this study relate to a priority-setting context only, since previous work indicates the profile BWS to be a feasible method for valuing health states [4], [7], [22]. Further qualitative research and replication of this study in a larger comparative study is important to explore and test these possible phenomena and reveal whether they are specific to the context of priority-setting in health care, or apply more broadly.

In response to the third objective, the DCE would appear to be the most acceptable method for most but not all participants in this sample, principally due to its presentation of whole profiles and ability to allow comparison between alternative profiles. There was some indication that the BWS was considered less burdensome than the DCE, but this would appear to be the case for only a minority in our sample. Although we did not test a BWS format which elicited a complete ranking of attribute/levels within each profile, further work is needed to confirm the purported benefits of BWS in terms of reduced participant burden. Again, findings of this study apply to a priority-setting context, but may not be generalisable to other contexts such as health state valuation. Nevertheless, whilst it is possible that different participant groups or different decision contexts might engender different task preferences; to our knowledge this is the first study internationally to compare the acceptability of these two tasks for participants in any context.

The few previous empirical studies that have compared a single profile case BWS task with a traditional format DCE have not compared the internal validity or consistency of the tasks, nor have they reported the comparative acceptability of the tasks for participants [4], [13], [14]. Thus, this qualitative exploration makes an important contribution to our understanding of the comparative merits of these methods. Our finding that participants on average preferred and were more consistent with responses to the traditional DCE is somewhat inconsistent with previous claims that the profile case BWS is likely to be less burdensome than the traditional DCE [8]. It is also incongruent with previous empirical studies suggesting participants exhibit a greater level of certainty in their decision-making with the profile case BWS task [13], [14], highlighting the need to explore the comparative merits of these two methods further.

Participants in this study were recruited from a University campus; therefore, we cannot be sure that the findings from this study would translate to other populations. Confirmation of the themes identified here is required for more diverse and larger samples. Despite this potential limitation, the study provides valuable insights into the approach participants take to the traditional DCE and profile case BWS choice tasks, the validity of the methods, and the acceptability of the tasks for participants. The findings also identify some clear areas for further research. A larger comparative study is currently being undertaken using this survey within a population based sample to compare the consistency of the preference structure indicated by models of DCE and BWS data, and to explore a number of the issues raised in this study further including response inconsistency and randomness of decision-making.

In conclusion, this study provides a further example of the value of employing established qualitative methods to address methodological questions within the preference elicitation literature. The findings support the validity of the DCE as a method to assess preferences in the priority-setting context of HTA decision criteria and for this context and sample, the DCE was found to be the most acceptable task for the majority of participants. The findings relating to the application of profile case BWS methods are less definitive than for the traditional DCE choice task, but suggest some inconsistency with previous claims that profile case BWS is likely to be less burdensome than a traditional format DCE task, in this priority setting context. However, important insights and avenues for future research to further clarify the comparative merits of the DCE and BWS preference elicitation methods have been identified.
